# Association of Nighttime Masked Uncontrolled Hypertension With Left Ventricular Hypertrophy and Kidney Function Among Patients with Chronic Kidney Disease Not Receiving Dialysis

**DOI:** 10.1001/jamanetworkopen.2022.14460

**Published:** 2022-05-26

**Authors:** Xiaocen Fu, Hong Ren, Jingyuan Xie, Weiming Wang, Yan Li, Pingjin Gao, Nan Chen

**Affiliations:** 1 Department of Nephrology, Institute of Nephrology, Ruijin Hospital, Shanghai Jiao Tong University School of Medicine, Shanghai, People’s Republic of China; 2 Department of Cardiovascular Medicine, Shanghai Key Laboratory of Hypertension, National Key Laboratory of Medical Genomics, The Shanghai Institute of Hypertension, Ruijin Hospital Affiliated to Shanghai Jiaotong University School of Medicine, Shanghai, People’s Republic of China

## Abstract

**Importance:**

Nighttime hypertension is prevalent and associated with adverse outcomes in patients with chronic kidney disease (CKD), but nighttime hypertension, a subtype of masked uncontrolled hypertension (MUCH), is often undetected among patients with controlled office blood pressure. Little attention has been paid to patients with CKD and nighttime MUCH.

**Objective:**

To investigate the prevalence of nighttime MUCH and its associations with cardiovascular and kidney outcomes in patients with CKD who were not receiving dialysis.

**Design, Setting, and Participants:**

This retrospective cohort study included patients with nondialysis CKD and hypertension, enrolled in Shanghai, China, from July 2012 through November 2020 and followed up for a median of 39 months.

**Exposures:**

Participants were classified as having controlled hypertension, sustained hypertension, and MUCH, which was further divided into isolated nighttime MUCH and day-night MUCH, assessed by office and ambulatory blood pressure monitoring.

**Main Outcomes and Measures:**

Left ventricular hypertrophy (LVH) was determined by echocardiography. The composite kidney outcome consisted of end-stage kidney diseases (ESKD) and a reduction of estimated glomerular filtration rate (eGFR) by 50% or more. Logistic and Cox regression assessed the associations of hypertension subtypes with LVH and kidney outcomes.

**Results:**

The 675 patients (425 [63.0%] men; mean [SD] age, 50.8 [15.9] years; mean [SD] eGFR, 61.6 [29.4] mL/min/1.73 m^2^) included 125 (19.3%) with controlled hypertension, 244 (37.6%) with MUCH, and 280 (43.1%) sustained hypertension. Among patients with MUCH, 2 (0.8%) had isolated daytime MUCH, 154 (63.1%) had isolated nighttime MUCH, and 88 (36.1%) had day-night MUCH. During a median (IQR) follow-up of 39 (19-64) months, 130 composite kidney events, including 97 ESKD events, occurred. Compared with controlled hypertension, MUCH and sustained hypertension were associated with LVH (eg, MUCH: odds ratio [OR], 2.94; 95% CI, 1.18-7.34; *P* = .02) and the composite kidney outcome (eg, MUCH: hazard ratio [HR], 4.12; 95% CI, 1.75-9.73; *P* = .001) after adjustment for age, sex, proteinuria, eGFR, and other baseline risk factors. Multivariate-adjusted associations were also significant between day-night MUCH and LVH (OR, 3.26; 95% CI, 1.15-9.25) and between isolated nighttime MUCH and the composite kidney outcome (HR, 4.27; 95% CI, 1.69-10.77).

**Conclusions and Relevance:**

In this cohort study, nighttime MUCH was common and associated with LVH and poor kidney outcomes among patients with hypertension and nondialysis CKD. These findings suggest that ambulatory blood pressure monitoring was inadequately used in patients with CKD and hypertension, calling for more widespread use, even in patients with controlled office hypertension.

## Key Points

**Question:**

Is nighttime masked uncontrolled hypertension (MUCH) in patients with chronic kidney disease (CKD) not receiving dialysis associated with worse cardiovascular and kidney outcomes?

**Findings:**

In this cohort study of 675 patients with hypertension and nondialysis CKD, approximately one-third of patients had nighttime MUCH, consisting of 13.0% day-night MUCH and 22.8% isolated nighttime MUCH. Day-night MUCH was associated with left ventricular hypertrophy, and isolated nighttime MUCH was associated with kidney function deterioration.

**Meaning:**

In this study, ambulatory blood pressure monitoring was necessary among patients with CKD to promote the management of nighttime hypertension and to benefit cardiovascular and kidney outcomes, even in patients with controlled office hypertension.

## Teaser

This cohort study investigates the prevalence of nighttime masked uncontrolled hypertension and its associations with cardiovascular and kidney outcomes in patients with chronic kidney disease (CKD) who were not receiving dialysis.

## Introduction

Chronic kidney disease (CKD) is a major public health problem, affecting 10.8% of the Chinese population.^1^ Hypertension is a common complication in patients with CKD, with a prevalence of 65% in patients with normal or nearly normal kidney function and up to 95% in patients with end stage kidney disease (ESKD).^2^ Given that hypertension is a strong risk factor for incident cardiovascular diseases, progression of kidney diseases, and mortality in the CKD population,^3,4^ strict blood pressure (BP) intervention and monitoring are needed to slow CKD progression and reduce cardiovascular risk.

The combined application of ambulatory BP monitoring (ABPM) and office BP measurement is increasingly recommended in making diagnosis and treatment decisions according to hypertension guidelines.^5,6^ ABPM helps to detect out-of-office hypertension, especially nighttime hypertension, which is the most important contribution of ABPM.^7^ Nighttime hypertension is reported to be of high prevalence and associated with adverse outcomes among patients with CKD,^8,9,10,11,12^ who could be misdiagnosed if management of hypertension is based on office BP measurements alone.

Masked uncontrolled hypertension (MUCH) has usually been defined as uncontrolled 24-hour or daytime hypertension in the presence of a controlled office BP.^13,14,15,16,17,18,19^ Recently, it was reported that the prevalence of nighttime masked hypertension among US adults was 18.8% to 22.7%.^20^ However, a similar estimate for patients with CKD is lacking. Additionally, the association between nighttime MUCH and cardiovascular and kidney outcomes among patients with CKD is unknown. Therefore, we conducted a retrospective analysis in Chinese patients with CKD not receiving dialysis.

## Methods

### Participants

From July 1, 2012, through November 31, 2020, patients with CKD and hypertension were enrolled in the Nephrology Department of Ruijin Hospital, which is affiliated with the Shanghai Jiao Tong University School of Medicine. The study protocol was approved and an exemption of informed consent granted because of the retrospective nature of the study by the Ethics Committee of the Ruijin Hospital. This study followed the Strengthening the Reporting of Observational Studies in Epidemiology (STROBE) reporting guideline.

**Figure 1.**
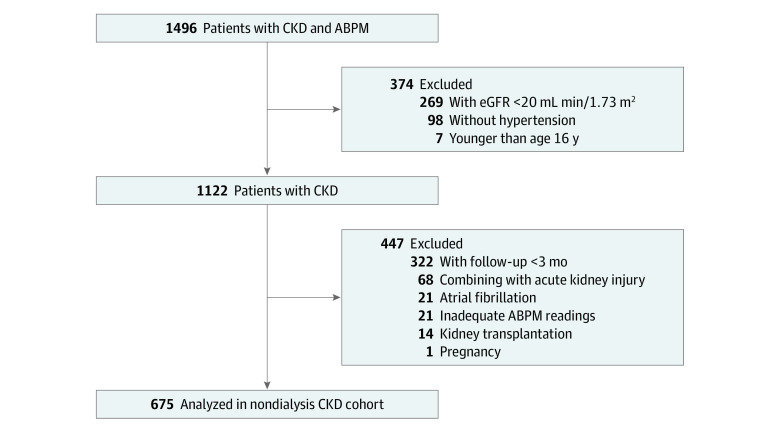
Study Flow Diagram ABPM indicates ambulatory blood pressure monitoring; CKD, chronic kidney disease; and eGFR, estimated glomerular filtration rate.

Inclusion criteria were age 16 years or older; diagnosis of CKD with markers of kidney damage or a glomerular filtration rate (GFR) of 60 mL/min/1.73 m^2^ or less for at least 3 months according to the Kidney Disease: Improving Global Outcomes (KDIGO) 2012 clinical practice guideline^21^; with an estimated GFR (eGFR) of at least 20 mL/min/1.73 m^2^ at baseline; and a diagnosis of hypertension based on an office systolic BP measurement of at least 140 mm Hg and diastolic BP measurement of at least 90 mm Hg or the use of antihypertensive drugs. Patients were excluded for having acute kidney injury; receiving kidney transplantation; being pregnancy; having atrial fibrillation; having inadequate ABPM readings; and having invalid follow-up data (ie, duration of follow up <3 months). A total of 1122 patients fulfilled the inclusion criteria, and 125 patients were excluded because of acute kidney injury (n = 68), kidney transplantation (n = 14), pregnancy (n = 1), atrial fibrillation (n = 21), and inadequate ABPM readings (n = 21). Among the remaining 997 patients with nondialysis CKD, 322 with a follow-up of less than 3 months without any kidney events were further excluded to avoid any possible confounding associated with the short follow-up. Therefore, 675 patients were included in the present analyses (Figure 1). Compared with the 322 excluded patients, the included cohort had fewer men, reported greater use of angiotensin-converting enzyme inhibitors or angiotensin receptor blockers, and had different CKD etiology as well as higher fasting glucose levels and ABPM values (eTable 1 in the Supplement).

### BP Measurement

Office BP was measured with an automated sphygmomanometer (Omron HBP-1300) after patients had 5 minutes of rest in a sitting position. Three consecutive readings with an interval of 1 minute were obtained and averaged for analyses. The 24-hour ABPM was performed with the 90217A monitors (Spacelabs Inc). The monitors recorded BP every 15 to 20 minutes from 6 am to 10 pm and every 30 minutes from 10 pm to 6 am. Daytime and nighttime were defined accordingly. If at least 1 systolic and diastolic BP measurement was obtained every hour, with at least 20 readings during the day and 7 readings during the night, the ABPM was considered adequate. The BP dipping ratio was calculated as the percentage of the difference between daytime and nighttime average BPs to the daytime average BP.

### Left Ventricular Hypertrophy

Echocardiography was performed by experienced cardiologists. End-diastolic interventricular septal wall thickness (IVST), left ventricular diameter (LVEDD), and posterior wall thickness (PWT) were assessed from M-mode tracings. Left ventricular mass (LVM)^22^ was calculated as 0.8 × 1.04 × ([LVEDD + IVST + PWT]^3^ – LVEDD^3^] + 0.6. The LVM index (LVMI) was the ratio of LVM to body surface area. Left ventricular hypertrophy (LVH) was defined as an LVMI greater than 115 g/m^2^ in men and greater than 95 g/m^2^ in women.^23^

### Kidney Assessment and Definition of Kidney Outcomes

Serum creatinine was analyzed by automated enzymatic methods. The eGFR was estimated using the CKD–Epidemiology Collaboration (CKD-EPI) formula and graded to CKD stages 1, 2, 3, and 4 according to KDIGO.^24^ We evaluated a composite kidney end point consisting of ESKD (ie, initiation of kidney replacement therapy) and a reduction of eGFR by 50% or greater, whichever occurred first. The occurrence of events was confirmed through medical records during follow-up visits.

### Definitions of Hypertension and Its Subtypes

As shown in the eTable 2 in the Supplement, office, daytime, and nighttime hypertension were defined as an average systolic BP of 140 mm Hg or greater and diastolic BP of 90 mm Hg or greater in the office, systolic BP of 135 mm Hg or greater and diastolic BP of 85 mm Hg or greater during the day, and systolic BP of 120 mm Hg or greater and diastolic BP of 70 mm Hg or greater during the night, based on the 2018 Chinese hypertension guideline,^6^ respectively. Controlled out-of-office hypertension was defined as controlled daytime and nighttime hypertension. White coat hypertension was uncontrolled office hypertension with controlled out-of-office hypertension. MUCH was controlled office hypertension with uncontrolled out-of-office hypertension. According to the 2017 American College of Cardiology/American Heart Association (ACC/AHA) hypertension guidelines,^5^ we defined stage 1 MUCH as a daytime systolic BP of 135 to 144 mm Hg and diastolic BP of 85 to 89 mm Hg or nighttime systolic BP of 120 to 139 mm Hg and diastolic BP of 70 to 84 mm Hg. Stage 2 MUCH was defined as a daytime systolic BP of 135 mm Hg or greater and diastolic BP of 85 mm Hg or greater or nighttime systolic BP of 140 mm Hg or greater and diastolic BP of 85 mm Hg or greater. MUCH was further subdivided into isolated daytime MUCH, isolated nighttime MUCH, and day-night MUCH (eTable 2 in the Supplement). Controlled hypertension and sustained hypertension were consistently controlled or uncontrolled BPs, respectively, both office and out-of-office.

### Statistical Analysis

Statistical analyses were performed using SPSS statistical software version 25 (IBM Corp). Data were tested for normal distribution using the Shapiro-Wilk test. Descriptive statistics are mean and SD for continuous variables, median and IQR for nonparametric variables, and frequency and percentage for categorical variables. Comparisons among groups were performed using nonparametric tests or analysis of variance with Bonferroni method for post hoc multiple comparisons for continuous variables and χ^2^ test for categorical variables. Univariate and multivariate logistic regression analyses were performed to explore factors associated with hypertension subtypes and to calculate odd ratios (ORs) and 95% CIs of LVH. Univariate and multivariate Cox regression analyses were performed to estimate the associations of hypertension subtypes with kidney outcomes. In multivariate analyses, age, sex, and body mass index were included as covariables in model 1 and diabetes, use of angiotensin-converting enzyme inhibitors or angiotensin receptor blockers, eGFR, hemoglobin level, and proteinuria were included in model 2. All *P* values were 2-sided and were considered significant if *P* < .05. Graphs were generated with Prism version 9 (GraphPad Software).

## Results

### Baseline Characteristics of Participants

Of the 675 patients with nondialysis CKD and hypertension (Table), 425 (63.0%) were men, 640 (94.8%) were receiving antihypertension treatment, 220 (32.6%) had a history of diabetes, 401 (59.4%) had glomerulonephritis, 34 (4.9%) had diabetic nephropathy, 52 (7.7%) had hypertensive nephropathy, and 189 (28.0%) had other causes of kidney disease. The mean (SD) age was 50.8 (15.9) years. The mean (SD) eGFR was 61.6 (29.4) mL/min/1.73 m^2^. Among all patients, 141 (20.9%), 169 (25.0%), 256 (37.9%), and 109 (16.1%) had CKD stages 1, 2, 3, and 4, respectively. The median (IQR) of 24-hour urinary protein excretion was 1786 (574-3938) mg.

**Table.** Baseline Characteristics of the Study Patients

Characteristic	Patients, No. (%)				*P* value	*P* value^b^	*P* value^c^
	Total (N = 675)^a^	Hypertension					
		Controlled (n = 125 [19.3%])	Masked uncontrolled (n = 244 [37.6%])	Sustained (n = 280 [43.1%])			
Age, mean (SD), y	50.8 (15.9)	44.3 (17.6)	49.5 (14.4)	54.6 (15.3)	<.001	.007	<.001
Sex							
Male	425 (63.0)	75 (60.0)	147 (60.2)	188 (66.8)	.22	NA	NA
Female	250 (37.0)	50 (40.0)	97 (39.2)	92 (33.2)			
BMI, mean (SD)	25.3 (4.0)	24.4 (3.6)	25.0 (3.7)	26.0 (4.3)	<.001	.44	<.001
Diabetes	220 (32.6)	21 (16.8)	66 (27.0)	220 (44.6)	<.001	NA	NA
Etiology of CKD							
Primary glomerulonephritis	401 (59.4)	94 (75.2)	161 (66.0)	132 (47.1)	<.001	NA	NA
Diabetic nephropathy	33 (4.9)	0 (0.0)	8 (3.3)	24 (8.6)			
Hypertensive nephropathy	52 (7.7)	8 (6.4)	17 (7.0)	24 (8.6)			
Others	189 (28.0)	23 (18.4)	58 (23.8)	100 (35.7)			
Antihypertensive agent use							
Use of drugs	640 (94.8)	125 (100.0)	222 (91.0)	269 (96.1)	<.001	NA	NA
No. of drugs, mean (SD)	2.0 (1.2)	1.5 (0.8)	1.7 (1.1)	2.4 (1.3)	<.001	.13	<.001
Laboratory measurements							
Fasting glucose, mean (SD), mg/dL	86.5 (21.6)	82.9 (14.4)	84.7 (18.02)	90.1 (27.0)	<.001	.96	.005
Serum albumin, mean (SD), g/dL	3.2 (0.8)	3.4 (0.8)	3.2 (0.9)	3.2 (0.8)	.06	.21	.06
Total cholesterol, median (IQR), mg/dL	201.1(170.1-251.4)	193.3(162.4-243.6)	201.1(174.0-251.4)	201.1(166.3-255.2)	.13	.46	.77
Serum creatinine, mean (SD), mg/dL	1.5 (0.7)	1.3 (0.6)	1.4 (0.6)	1.6 (0.7)	<.001	.14	<.001
eGFR, mean (SD), ml/min/1.73m^2^	61.6 (29.4)	74.0 (31.3)	65.3 (30.3)	52.4 (24.8)	<.001	.02	<.001
Proteinuria, median (IQR), mg/24h	1786(574-3938)	1337 (403-2996)	1627 (552-3616)	2232 (840-5425)	<.001	.09	<.001
Hemoglobin, mean (SD), g/dL	12.5 (2.1)	13.0 (2.1)	12.4 (2.2)	12.3 (2.0)	.004	.03	.003
LVMI, median (IQR), g/m^2^	85.1 (73.4-102.6)	76.6 (66.0-87.2)	82.0 (72.0-97.2)	94.5 (79.9-110.5)	<.001	.002	<.001
**BP parameter, mean (SD), mm Hg**							
Office BP							
Systolic	138.3 (17.8)	121.8 (9.9)	127.6 (8.3)	154.2 (13.1)	<.001	<.001	<.001
Diastolic	81.1 (10.3)	73.0 (6.9)	77.7 (6.6)	87.3 (10.6)	<.001	<.001	<.001
24-h BP							
Systolic	129.8 (16.2)	111.8 (8.6)	126.1 (10.5)	142.2 (13.3)	<.001	<.001	<.001
Diastolic	79.4 (9.7)	69.7 (5.5)	80.3 (7.1)	83.8 (9.9)	<.001	<.001	<.001
Daytime BP							
Systolic	130.6 (16.1)	113.4 (9.1)	126.4 (10.4)	142.9 (13.4)	<.001	<.001	<.001
Diastolic	80.1 (9.7)	71.0 (5.9)	80.7 (7.5)	84.3 (10.1)	<.001	<.001	<.001
Nighttime BP							
Systolic	125.4 (19.8)	102.8 (7.9)	123.9 (14.9)	138.5 (17.0)	<.001	<.001	<.001
Diastolic	75.8 (11.4)	62.5 (4.8)	78.1 (8.2)	80.8 (11.0)	<.001	<.001	<.001
BP dipping rate, mean (SD), %							
Systolic	4.0 (8.4)	9.1 (6.3)	2.0 (8.4)	3.0 (8.1)	<.001	<.001	<.001
Diastolic	5.4 (8.7)	11.7 (6.9)	2.9 (8.7)	4.1 (7.9)	<.001	<.001	<.001

Abbreviations: BMI, body mass index (calculated as weight in kilograms divided by height in meters squared); BP, blood pressure; CKD, chronic kidney disease; eGFR, estimated glomerular filtration rate; LVMI, left ventricular hypertrophy index.

SI conversion factors: To convert fasting glucose to millimoles per liter, multiply by 0.0555; albumin to grams per liter, multiply by 10; total cholesterol to millimoles per liter, multiply by 0.0259; serum creatinine to micromoles per liter, multiply by 88.4; hemoglobin to grams per liter, multiply by 10.

^a^ Among the total 675 patients, 26 had white-coat hypertension and were omitted from this table.

^b^ Controlled hypertension vs masked uncontrolled hypertension.

^c^ Controlled hypertension vs sustained hypertension.

As shown in the Table, a total of 125 (19.3%), 244 (37.6%), and 280 (43.1%) had controlled hypertension, MUCH, and sustained hypertension, respectively. The 26 patients with white coat hypertension were excluded from further analyses because of limited sample size. Compared with patients with controlled hypertension, patients with MUCH and sustained hypertension were older, more likely to have diabetes, had lower hemoglobin levels and eGFR, had higher levels of proteinuria and office and ambulatory BP values, and used more antihypertensive drugs. The 178 patients with stage 1 MUCH (73.0%) had similar characteristics as the 66 patients with stage 2 MUCH (27.0%), except for the ambulatory BP levels and dipping rates (eTable 3 in the Supplement). eTable 4 in the Supplement provides more detailed information on antihypertensive agent use in the study participants.

**Figure 2.**
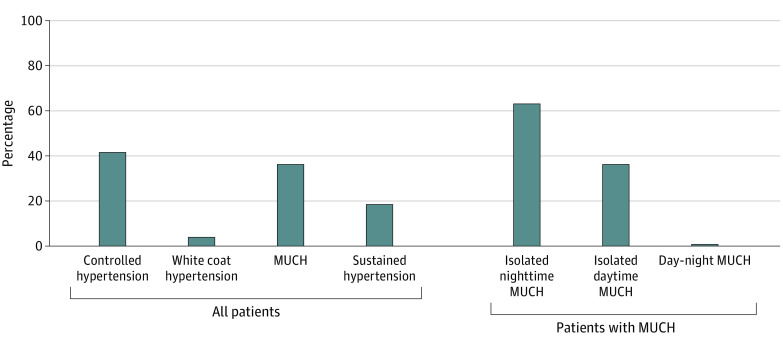
Prevalence of Subtypes of Hypertension and Masked Uncontrolled Hypertension (MUCH)

Among the 244 patients with MUCH, isolated daytime, isolated nighttime, and day-night MUCH accounted for 2 patients (0.8%), 154 patients (63.1%), and 88 patients (36.1%), respectively (Figure 2). The 2 patients with isolated daytime MUCH were excluded from further analyses because of limited sample size. Patients with isolated nighttime MUCH had similar clinical characteristics as those with day-night MUCH, except for having lower office and ambulatory BP measurements (eTable 5 in the Supplement).

### LVH by Hypertension Subtype

**Figure 3.**
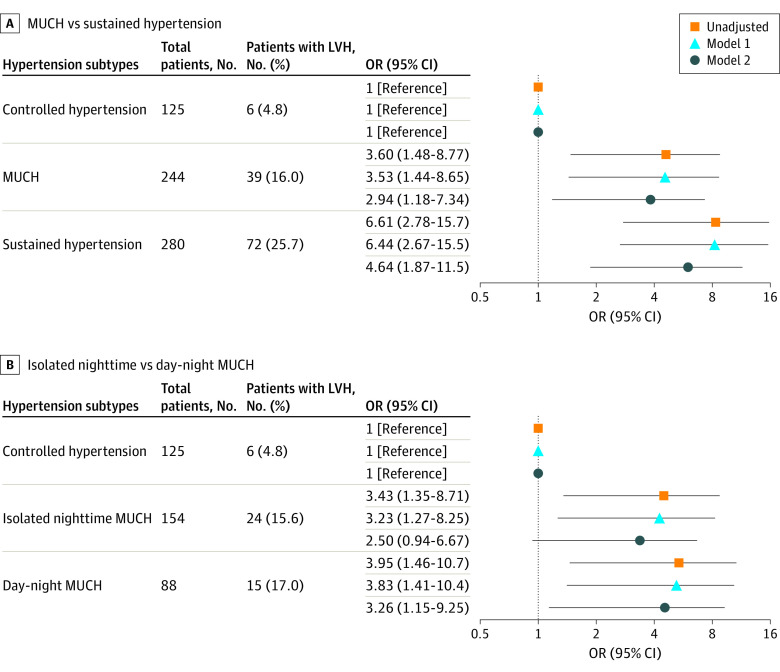
Association Between Left Ventricular Hypertrophy (LVH) and Hypertension Subtypes Model 1 adjustment variables included age, sex, and body mass index. Model 2 adjustment variables included age, sex, body mass index, diabetes, use of angiotensin-converting enzyme inhibitors or angiotensin receptor blockers, estimated glomerular filtration rate, hemoglobin, and proteinuria. MUCH indicates masked uncontrolled hypertension; and OR, odds ratio.

LVH was detected in 6 patients with controlled hypertension (5.2%), 39 patients with MUCH (16.5%), and 72 patients with sustained hypertension (26.7%); it was detected among 24 patients with isolated nighttime MUCH (15.9%) and 15 with day-night MUCH (17.9%). Figure 3 shows unadjusted and adjusted ORs of LVH associated with the hypertension subtypes. Compared with controlled hypertension, MUCH (OR, 2.94; 95% CI, 1.18-7.34; *P* = .02) and sustained hypertension (OR, 4.63; 95% CI, 1.87-11.49; *P* = .001) were associated with higher odds of LVH in fully adjusted analyses. In the further fully adjusted grade analysis, stage 2 MUCH was associated with LVH with an OR of 4.03 (95% CI, 1.38-11.79; *P* = .009) (eTable 6 in the Supplement).

Among patients with MUCH, day-night hypertension was significantly associated with increased odds of LVH compared with controlled hypertension in fully adjusted analyses (OR, 3.26; 95% CI, 1.15-9.25; *P* = .03). The odds for isolated nighttime MUCH was not significant (OR, 2.50; 95% CI, 0.94-6.67; *P* = .07) after fully multivariable adjustment (Figure 3).

Furthermore, patients with diabetic nephropathy and hypertensive nephropathy had higher LVMI than patients with primary glomerulonephritis and other etiology (eTable 7 in the Supplement). However, after additional adjustment including etiology of CKD into model 2, associations of hypertension and MUCH subtypes with LVH (eTable 8 in the Supplement) and kidney composite outcomes (eTable 9 in the Supplement) remained unaltered.

### Kidney Outcomes by Hypertension Subtype

**Figure 4.**
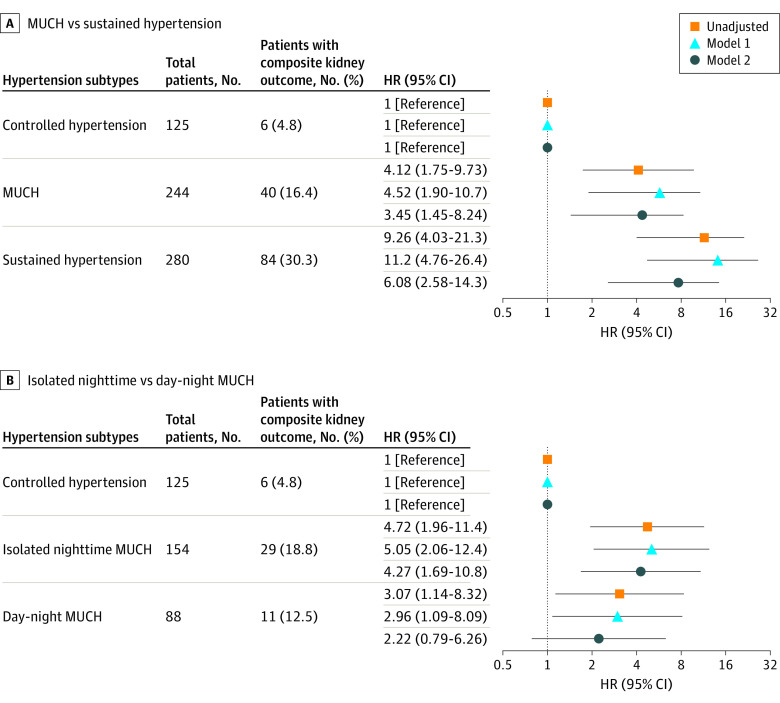
Association Between Composite Kidney Outcomes and Hypertension Subtypes Model 1 adjustment variables included age, sex, and body mass index. Model 2 adjustment variables included age, sex, body mass index, diabetes, use of angiotensin-converting enzyme inhibitors or angiotensin receptor blockers, estimated glomerular filtration rate, hemoglobin, and proteinuria. HR indicates hazard ratio; and MUCH, masked uncontrolled hypertension.

During a median (IQR) follow-up of 39 (19-64 months) month, 130 (19.3%) composite kidney events and 97 (14.4%) ESKD events occurred. Event numbers and hazard ratios (HRs) with 95% CIs by hypertension subtype are reported in Figure 4 for the composite kidney outcome and in the eFigure in the Supplement for ESKD.

In the univariate analyses, compared with controlled hypertension, MUCH (HR, 4.12; 95% CI, 1.75-9.73; *P* = .001) and sustained hypertension (HR, 9.26; 95% CI, 4.03-21.30; *P* < .001) were significantly associated with increased risk of the composite kidney outcome and ESKD. The significance persisted after partial and full adjustment for covariates, except for the association of ESKD with MUCH after full multivariable adjustment (eg, day-night MUCH and LVH: OR, 3.26; 95% CI, 1.15-9.25; isolated nighttime MUCH and composite kidney outcome: HR, 4.27; 95% CI, 1.69-10.77). Furthermore, both stages 1 and 2 MUCH had a significantly higher risk of composite kidney outcomes compared with controlled hypertension (eTable 10 in the Supplement).

Among patients with MUCH, isolated nighttime MUCH also had significantly higher risk of composite kidney outcomes than controlled hypertension in unadjusted analyses as well as partly and fully adjusted analyses. Similar results were observed for patients with day-night MUCH and for the associations between MUCH and ESKD, although the HRs became nonsignificant after full multivariable adjustment.

### Sensitivity Analysis

Using the thresholds of office BP measurement (systolic BP, 130 mm Hg; diastolic BP, 80 mm Hg), daytime BP measurement (systolic BP, 130 mm Hg; diastolic BP, 80 mm Hg) and nighttime BP measurement (systolic BP, 110 mm Hg; diastolic BP, 65 mm Hg) recommended by the 2017 ACC/AHA hypertension guidelines, a total of 74 (11.0%), 107 (15.9%), and 440 (65.2%) had controlled hypertension, MUCH, and sustained hypertension, respectively. Among the 107 patients with MUCH, isolated daytime, isolated nighttime, and day-night MUCH accounted for 10 (9.3%), 53 (49.6%), and 44 (41.1%), respectively. Compared with controlled hypertension, MUCH was not associated with a statistically significant higher odds of LVH (OR, 2.62; 95% CI, 0.70-9.76) (eTable 11 in the Supplement), but it was associated with increased risks of the composite kidney outcome (HR, 4.78; 95% CI, 1.09-21.03) (eTable 12 in the Supplement).

## Discussion

The present study is the first investigation with follow-up data focused on nighttime MUCH in patients with CKD, to our knowledge. Our results suggest that MUCH, especially nighttime MUCH, was common in patients with nondialysis CKD who also have hypertension and was associated with LVH and the progression of kidney diseases.

According to current guidelines, nighttime BP control should be an important part of hypertension management, especially in Asia.^25,26,27^ Observational studies in general populations and patients with hypertension and CKD showed that elevated nighttime BP was associated with target organ damage.^28^ A multicenter cohort study of 356 older Chinese patients with CKD showed that proteinuria and kidney progression was associated with the lowest level of nighttime BP.^29^ In a cross-sectional analysis among 2386 patients with nondialysis CKD, patients with isolated nighttime hypertension had higher odds of target organ damage, including LVH, abnormal carotid intima-media thickness, low eGFR, and albuminuria.^28^

Diagnosis of nighttime hypertension relies on ABPM, meaning that nighttime hypertension may often be masked by normal office BP measurements. In this study, MUCH accounted for 37.6% of patients with nondialysis CKD, a little higher than the prevalence range of 14.5% to 33.9% observed in prior studies.^13,16,30,31^ Different definitions of MUCH may partly explain the different prevalence observed across the studies. In most prior studies, uncontrolled out-of-office hypertension was based on the 24-hour BP measurement only, meaning that uncontrolled nighttime hypertension was masked by controlled daytime hypertension and vice versa. The BP dipping rate in the MUCH group was lower than that in the sustained hypertension group, suggesting nighttime hypertension was more often ignored among patients with controlled office hypertension in our clinical practice.

As a major type of MUCH, nighttime MUCH is a cause for concern. We observed that nearly all patients with MUCH had nighttime hypertension, and isolated nighttime hypertension accounted for 63.1% of patients. Overall, 34.4% of patients with nondialysis CKD had nighttime MUCH, suggesting that more than one-third of patients with CKD and uncontrolled nighttime hypertension would be misdiagnosed and miss treatment if only relying on office BP measurements. Similarly, a US population-based analysis reported that 27.9% (95% CI, 22.9%-33.0%) participants in the CKD subgroup had masked asleep hypertension.^20^ Among 738 White patients with treated hypertension and controlled clinic BP measurements, 48.5% patients had nighttime MUCH.^32^ Among 1808 untreated outpatients recruited from a hypertension clinic, Zhang et al^33^ reported that 11.6% patients had isolated nighttime hypertension with increased carotid intima-media thickness and urinary albumin-to-creatinine ratio compared with patients without hypertension.

MUCH was associated with kidney dysfunction, the extent of proteinuria, and higher LVMI in earlier cohorts.^13,18,31,34,35^ In the current study, both stage 1 and 2 MUCH were associated with LVH and an increased risk of kidney function deterioration. Furthermore, when using lower BP thresholds recommended by the 2017 ACC/AHA hypertension guidelines, MUCH remained significant in the association with kidney function deterioration. A cross-sectional and observational cohort that enrolled 1492 patients with CKD and an eGFR of 20 to 70 mL/min/1.73 m^2^ reported that MUCH was associated with lower eGFR and greater LVMI only in the presence of elevated nighttime BP,^16^ suggesting that nighttime hypertension played an important role in target organ damage in patients with MUCH. In this study, we verified that nighttime MUCH, irrespective of the combination with daytime MUCH, was associated with LVH. Furthermore, we reported, to our knowledge for the first time, that nighttime MUCH increased the risk of progression of kidney diseases and ESKD in patients with CKD. Most previous studies evaluating the prognostic value of MUCH relied on daytime BP measurements or the 24-hour BP measurement.^18,36,37,38,39,40,41^ Only a few emphasized nighttime BP.^18,36,37,38,39,40,41^ In 738 White patients with treated hypertension, nighttime MUCH was associated with increased risk of cardiovascular events, but isolated nighttime MUCH did not attain statistical significance after adjustment.^32^ A report from the Chronic Renal Insufficiency Cohort Study demonstrated that MUCH, defined as achieved office BP and elevated 24-hour BP, was associated with high risk of cardiovascular disease and progression of kidney disease in patients with CKD, and nighttime BP predicted the progression of kidney disease.^39^

We observed that more than one-third patients with CKD had MUCH, with 99.5% with nighttime MUCH, suggesting the poor management status of nighttime hypertension in our clinical practice. It is important to note that patients with nighttime MUCH, irrespective of the subtype (day-night or isolated nighttime), were at high risk of target organ damage and kidney disease progression, reinforcing the importance of controlling nighttime BP and both subtypes of nighttime MUCH to prevent poor kidney outcomes in patients with CKD. Our findings support the value of 24-hour ABPM in the management of hypertension, especially in patients with CKD. However, clinical use of ABPM has been limited in the past by variable factors, including economic and human cost. Meanwhile, management of nighttime MUCH still lacks clinical experience reports or evidence-based medical research. For example, antihypertensive regimens might be different for the 2 types of MUCH, with varying dosing times of drugs and choice of long or short half-life drugs. Future clinical trials are needed to explore effective interventions on nighttime MUCH and to assess the effect of lowering BP on clinical outcomes among patients with CKD.

### Limitations

This study has limitations, including its retrospective observational design. Second, the patients were recruited from a single Chinese nephrology ward in a tertiary hospital, among whom nearly 60% had glomerulonephritis. The incidence rate of ESKD was higher than that in other studies,^39,42,43^ with a similar baseline eGFR. Our conclusions may not be directly generalized to other populations. Third, ABPM was performed once at baseline. Considering the moderate reproducibility of ABPM,^44^ it is better to define MUCH based on repeated ABPM. Fourth, residual confounding or some unmeasured variables,^45^ such as interventions over follow-up, physical activities, diet, smoking, and sleep disorders, might in part explain the associations between MUCH and outcomes. Fifth, there might be a chance of false-positive results because of multiple comparisons in the analyses of logistic and Cox regressions. The small sample size in subgroups may affect the significance of differences. Prospective cohort studies with larger scale and including other ethnic populations may be required.

## Conclusions

The present study found that MUCH, especially nighttime MUCH, was common among patients with CKD and was associated with LVH and poor kidney outcomes. These findings may suggest the importance and irreplaceability of ABPM in the management of nighttime hypertension in patients with CKD.

## Notes

Supplement. **eTable 1.** Baseline Characteristics of Included Patients and Excluded Patients With Follow-up Less Than 3 Months **eTable 2.** Definitions of Hypertension and Its Subtypes **eTable 3.** Clinical Characteristics of Stage 1-2 Masked Uncontrolled Hypertension **eTable 4.** Use of Antihypertensive Agents in the Study Patients **eTable 5.** Clinical Characteristics of Masked Uncontrolled Hypertension Subtypes **eTable 6.** Association Between Left Ventricular Hypertrophy and Stage 1-2 Masked Uncontrolled Hypertension **eTable 7.** Left Ventricular Mass Index Among Different Etiology of Chronic Kidney Disease Patients **eTable 8.** Left Ventricular Hypertrophy Among Study Patients Additionally Adjusted by Etiology of Chronic Kidney Disease **eTable 9.** Composite Kidney Outcomes Among Study Patients Additionally Adjusted by Etiology of Chronic Kidney Disease **eTable 10.** Association Between Composite Kidney Outcomes and Stage 1-2 Masked Uncontrolled Hypertension **eTable 11.** Association Between Left Ventricular Hypertrophy and Hypertension Subtypes Based on 2017 ACC/AHA Hypertension Guidelines **eTable 12.** Association Between Composite Kidney Outcomes and Hypertension Subtypes Based on 2017 ACC/AHA Hypertension Guidelines **eFigure.** Association Between ESKD and Hypertension Subtypes
