# Supplementary material for: Association of Nighttime Masked Uncontrolled Hypertension With Left Ventricular Hypertrophy and Kidney Function Among Patients with Chronic Kidney Disease Not Receiving Dialysis
Source: JAMA Netw Open. 2022 May 26;5(5):e2214460. doi: 10.1001/jamanetworkopen.2022.14460 (PMC9136624; doi:10.1001/jamanetworkopen.2022.14460)
Supplement: Supplement. — eTable 1. Baseline Characteristics of Included Patients and Excluded Patients With Follow-up Less Than 3 Months eTable 2. Definitions of Hypertension and Its Subtypes eTable 3. Clinical Characteristics of Stage 1-2 Masked Uncontrolled Hypertension eTable 4. Use of Antihypertensive Agents in the Study Patients eTable 5. Clinical Characteristics of Masked Uncontrolled Hypertension Subtypes eTable 6. Association Between Left Ventricular Hypertrophy and Stage 1-2 Masked Uncontrolled Hypertension eTable 7. Left Ventricular Mass Index Among Different Etiology of Chronic Kidney Disease Patients eTable 8. Left Ventricular Hypertrophy Among Study Patients Additionally Adjusted by Etiology of Chronic Kidney Disease eTable 9. Composite Kidney Outcomes Among Study Patients Additionally Adjusted by Etiology of Chronic Kidney Disease eTable 10. Association Between Composite Kidney Outcomes and Stage 1-2 Masked Uncontrolled Hypertension eTable 11. Association Between Left Ventricular Hypertrophy and Hypertension Subtypes Based on 2017 ACC/AHA Hypertension Guidelines eTable 12. Association Between Composite Kidney Outcomes and Hypertension Subtypes Based on 2017 ACC/AHA Hypertension Guidelines eFigure. Association Between ESKD and Hypertension Subtypes [file jamanetwopen-e2214460-s001.pdf]

Supplemental Online Content

Fu X, Ren H, Xie J, et al. Association of nighttime masked uncontrolled hypertension with left ventricular hypertrophy and kidney function among patients with chronic kidney disease not receiving dialysis. *JAMA Netw Open.* 2022;5(5):e2214460. doi:10.1001/jamanetworkopen.2022.14460

eTable 1. Baseline Characteristics of Included Patients and Excluded Patients With Follow-up Less Than 3 Months

eTable 2. Definitions of Hypertension and Its Subtypes

eTable 3. Clinical Characteristics of Stage 1-2 Masked Uncontrolled Hypertension

eTable 4. Use of Antihypertensive Agents in the Study Patients

eTable 5. Clinical Characteristics of Masked Uncontrolled Hypertension Subtypes

eTable 6. Association Between Left Ventricular Hypertrophy and Stage 1-2 Masked Uncontrolled Hypertension

eTable 7. Left Ventricular Mass Index Among Different Etiology of Chronic Kidney Disease Patients

eTable 8. Left Ventricular Hypertrophy Among Study Patients Additionally Adjusted by Etiology of Chronic Kidney Disease

eTable 9. Composite Kidney Outcomes Among Study Patients Additionally Adjusted by Etiology of Chronic Kidney Disease

eTable 10. Association Between Composite Kidney Outcomes and Stage 1-2 Masked Uncontrolled Hypertension

eTable 11. Association Between Left Ventricular Hypertrophy and Hypertension Subtypes Based on 2017 ACC/AHA Hypertension Guidelines

eTable 12. Association Between Composite Kidney Outcomes and Hypertension Subtypes Based on 2017 ACC/AHA Hypertension Guidelines

eFigure. Association Between ESKD and Hypertension Subtypes

This supplemental material has been provided by the authors to give readers additional information about their work.

© 2022 Fu X et al. *JAMA Network Open.*

eTable 1. Baseline Characteristics of Included Patients and Excluded Patients with Follow-up Less than 3 Months

	Included	Excluded	***P*** value
N (%)	675	322	
Age, mean (SD), y	50.8 (15.9)	49.5 (16.2)	.83
Male, n (%)	425 (63.0)	226 (70.2)	.02
Female, n (%)	250 (37.0)	96 (29.8)	
BMI, mean (SD), kg/m2	25.3 (4.0)	25.1 (3.7	.36
Diabetes mellitus, n (%)	220 (32.6)	105(32.6)	.97
Etiology of CKD, n (%)			<.001
Primary glomerulonephritis	401 (59.4)	133 (41.2)	
Diabetic nephropathy	33 (4.9)	32 (10.0)	
Hypertensive nephropathy	52 (7.7)	34 (10.6)	
others, n (%)	189 (28.0)	123 (38.2)	
Antihypertensive agent use			
Use of drugs, n (%)	640 (94.8)	272 (84.5)	<.001
Number of drugs, mean (SD), n	2.0 (1.2)	1.9 (1.3)	.06
ACEI or ARB, n (%)	521 (77.2)	198 (61.5)	<.001
CCB, n (%)	418 (61.9)	189 (58.7)	.28
β-blockers, n (%)	187 (27.7)	84 (26.1)	.58
α-blockers, n (%)	124 (18.4)	66 (20.5)	.32
Diuretics, n (%)	38 (5.6)	24 (7.5)	.22
Laboratory measurements			
Fasting glucose, mean (SD), mg/dL	86.5 (21.6)	90.1 (30.6)	.004
Serum albumin, mean (SD), g/dL	3.2 (0.8)	3.2 (0.9)	.007
Total cholesterol, median (IQR), mg/dL	201.1 (170.1-251.4)	197.2 (158.5-247.5)	.28
Serum Cr, mean (SD), mg/dL	1.5 (0.7)	1.6 (0.7)	.61
eGFR, mean (SD), ml/min/1.73m2	61.6 (29.4)	59.3 (29.2)	.81
Proteinuria, median (IQR), mg/24h	1786 (574-3938)	1818 (562-4908)	.70
Hemoglobin, mean (SD), g/dL	12.4 (2.1)	12.3 (2.2)	.30
LVMI, median (IQR), g/m^2	85.1 (73.4-102.6)	88.7 (76.4-105.0)	.004
BP parameter			
Office SBP, mean (SD), mmHg	138.3 (17.8)	140.2 (16.3)	.11
Office DBP, mean (SD), mmHg	81.1 (10.3)	82.4 (10.2)	.94
24-h SBP, mean (SD), mmHg	129.8 (16.2)	132.3(15.2)	.07
24-h DBP, mean (SD), mmHg	79.4 (9.7)	81.3 (9.9)	.46
Daytime SBP, mean (SD), mmHg	130.6 (16.1)	133.7 (15.1)	.08
Daytime DBP, mean (SD), mmHg	80.1 (9.7)	81.9 (9.9)	.52
Nighttime SBP, mean (SD), mmHg	125.4 (19.8)	128.3 (18.2)	.05
Nighttime DBP, mean (SD), mmHg	75.8 (11.4)	78.0 (11.4)	.52
SBP dipping rate, mean (SD), %	4.0 (8.4)	4.1 (7.7)	.14
DBP dipping rate, mean (SD), %	5.4 (8.7)	4.8 (7.8)	.01

© 2022 Fu X et al. *JAMA Network Open.*

Values are means (standard deviations), median (quartile ranges) or number (percentage). SI conversion factors: To convert fasting glucose to mmol/L, multiply by 0.0555; albumin to g/L, multiply by 10; total cholesterol to mmol/L, multiply by 0.0259; serum creatinine to μmol/L, multiply by 88.4; hemoglobin to g/L, multiply by 10.

Abbreviations: CKD, chronic kidney disease; ACEI, angiotensin-converting enzyme inhibitor; ARB, angiotensin receptor blocker; CCB, calcium channel blocker; BMI, body mass index; eGFR, estimated glomerular filtration rate; Cr, creatinine; eGFR, estimated glomerular filtration rate; LVMI, left ventricular hypertrophy index; SBP, systolic blood pressure; DBP, diastolic blood pressure.

© 2022 Fu X et al. *JAMA Network Open.*

eTable 2. Definitions of Hypertension and Its Subtypes

	**Office Hypertension**	**Out-of-Office Hypertension**	
	Office BP ≥ 140/90 mmHg	Daytime BP ≥ 135/85 mmHg	Nighttime BP ≥ 120/70 mmHg
Controlled Hypertension	-	-	-
MUCH	-	+ or / and	+
Day-Night MUCH	-	+	+
Isolated Nighttime MUCH	-	-	+
Isolated Daytime MUCH	-	+	-
White Coat Hypertension	+	-	-
Sustained Hypertension	+	+ or / and	+

Note: -: Controlled; +: Elevated

Abbreviations: MUCH, masked uncontrolled hypertension.

© 2022 Fu X et al. *JAMA Network Open.*

eTable 3. Clinical Characteristics of Stage 1-2 Masked Uncontrolled Hypertension

	**Stage 1**	**Stage 2**	*P* value
N (%)	178 (73.0)	66 (27.0)	
Age, mean (SD), y	49.7 (13.9)	49.0 (15.7)	.76
Male, n (%)	112 (62.9)	35 (53.0)	.16
Female, n (%)	66 (37.1)	31 (47.0)	
BMI, mean (SD), kg/m2	25.1 (3.8)	24.5 (3.4)	.22
Diabetes mellitus, n (%)	49 (27.5)	17 (25.8)	.78
Etiology of CKD, n (%)			.20
Primary glomerulonephritis	117 (65.7)	44 (66.7)	
Diabetic nephropathy	5 (2.8)	1 (1.5)	
Hypertensive nephropathy	9 (5.1)	8 (12.1)	
others, n (%)	47 (26.4)	13 (19.7)	
Antihypertensive agent use			
Use of drugs, n (%)	163 (91.6)	59 (89.4)	.60
Number of drugs, mean (SD), n	1.7 (1.0)	1.8 (1.3)	.33
ACEI or ARB, n (%)	125 (70.2)	50 (75.8)	.39
CCB, n (%)	100 (56.2)	35 (53.0)	.66
β-blockers, n (%)	36 (20.2)	17 (25.8)	.35
α-blockers, n (%)	20 (11.2)	12 (18.5)	.15
Diuretics, n (%)	8 (4.5)	4 (6.1)	.62
Laboratory measurements			
Fasting glucose, mean (SD), mg/dL	84.7 (18.0)	82.9 (18.0)	.77
Serum albumin, mean (SD), g/dL	3.2 (0.8)	3.1 (0.9)	.20
Total cholesterol, median (IQR), mg/dL	201.9(173.2-242.1)	207.3(176.5-300.3)	.23
Serum Cr, mean (SD), mg/dL	1.4 (0.7)	1.4 (0.7)	.88
eGFR, mean (SD), ml/min/1.73m2	65.5 (30.5)	64.8 (29.9)	.88
Proteinuria, median (IQR), mg/24h	1527.0(470.1-3729.4)	1876.8(802.4-3585.3)	.18
Hemoglobin, mean (SD), g/dL	12.6 (2.1)	12.1 (2.3)	.14
LVMI, median (IQR), g/m^2	81.2(71.5-95.4)	86.0(76.3-107.0)	.04
BP parameter			
Office SBP, mean (SD), mmHg	127.2 (8.1)	128.6 (8.9)	.25
Office DBP, mean (SD), mmHg	77.4 (6.3)	78.6 (7.2)	.25
24-h SBP, mean (SD), mmHg	122.5 (8.4)	135.9 (9.3)	<.001
24-h DBP, mean (SD), mmHg	78.2 (5.7)	85.7 (7.8)	<.001
Daytime SBP, mean (SD), mmHg	123.1 (8.7)	135.3 (9.4)	<.001
Daytime DBP, mean (SD), mmHg	78.8 (6.2)	85.7 (8.3)	<.001
Nighttime SBP, mean (SD), mmHg	118.4 (9.5)	138.8 (16.5)	<.001
Nighttime DBP, mean (SD), mmHg	74.9 (5.1)	86.5 (8.8)	<.001
SBP dipping rate, mean (SD), %	3.7 (6.5)	-2.7 (11.0)	<.001
DBP dipping rate, mean (SD), %	4.6 (6.9)	-1.6 (11.3)	<.001

© 2022 Fu X et al. *JAMA Network Open.*

Values are means (standard deviations), median (quartile ranges) or number (percentage). SI conversion factors: To convert fasting glucose to mmol/L, multiply by 0.0555; albumin to g/L, multiply by 10; total cholesterol to mmol/L, multiply by 0.0259; serum creatinine to μmol/L, multiply by 88.4; hemoglobin to g/L, multiply by 10.

Abbreviations: CKD, chronic kidney disease; ACEI, angiotensin-converting enzyme inhibitor; ARB, angiotensin receptor blocker; CCB, calcium channel blocker; BMI, body mass index; eGFR, estimated glomerular filtration rate; Cr, creatinine; eGFR, estimated glomerular filtration rate; LVMI, left ventricular hypertrophy index; SBP, systolic blood pressure; DBP, diastolic blood pressure.

© 2022 Fu X et al. *JAMA Network Open.*

eTable 4. Use of Antihypertensive Agents in the Study Population

	Total*	Controlled Hypertension	Masked Uncontrolled Hypertension	Sustained Hypertension	*P* value
N (%)	675	125 (19.3)	244 (37.6)	280 (43.1)	
ACEI or ARB, n (%)	521 (77.2)	110 (88.0)	176 (72.1)	217 (77.5)	.002
CCB, n (%)	418 (61.9)	45 (36.0)	134 (54.9)	220 (78.6)	<.001
β-blockers, n (%)	187 (27.7)	18 (14.4)	53 (21.7)	112 (40.0)	<.001
α-blockers, n (%)	124 (18.4)	5 (4.0)	32 (13.1)	85 (30.4)	<.001
Diuretics, n (%)	38 (5.6)	3 (2.4)	12 (4.9)	22 (7.9)	.07

*Among the total 675 patients, 26 were white-coat hypertension and omitted from this table. Values are number (percentage).

Abbreviations: ACEI, angiotensin-converting enzyme inhibitor; ARB, angiotensin receptor blocker; CCB, calcium channel blocker.

© 2022 Fu X et al. *JAMA Network Open.*

eTable 5. Clinical Characteristics of Masked Uncontrolled Hypertension Subtypes

	Day-Night MUCH	Isolated Nighttime MUCH	*P* value
N (%)	88 (36.1)	154 (63.1)	
Age, mean (SD), y	49.2 (14.1)	49.6 (14.7)	.32
Male, n (%)	56 (63.6)	89 (57.8)	.30
Female, n(%)	32 (36.4)	65 (42.2)	
BMI, mean (SD), kg/m2	24.8 (3.7)	25.1 (3.7)	.82
Diabetes mellitus, n (%)	21 (23.9)	45 (29.2)	.05
Etiology of CKD, n (%)			.08
Primary glomerulonephritis	55 (62.5)	104 (67.5)	
Diabetic nephropathy	1 (1.1)	7 (4.5)	
Hypertensive nephropathy	5 (5.7)	12 (7.8)	
others, n (%)	27 (30.7)	31 (20.1)	
Antihypertensive agent use			
Use of drugs, n (%)	81 (92.0)	140 (90.9)	.10
Number of drugs, mean (SD), n	1.8 (1.1)	1.7 (1.1)	.96
ACEI or ARB, n (%)	52 (59.1)	111 (72.1)	.03
CCB, n (%)	64 (72.7)	81 (52.6)	.03
β-blockers, n (%)	22 (25.0)	31 (20.1)	.02
α-blockers, n (%)	16 (18.2)	16 (10.4)	.01
Diuretics, n (%)	3 (3.4)	9 (5.8)	.60
Laboratory measurements			
Fasting glucose, mean (SD), mg/dL	84.7 (19.8)	82.9 (18.0)	.63
Serum albumin, mean (SD), g/dL	3.1 (9.0)	3.2 (0.8)	.22
Total cholesterol, median (IQR), mg/dL	208.8(177.9-251.4)	201.1(174.0-251.4)	.50
Serum Cr, mean (SD), mg/dL	1.3 (0.6)	1.4 (0.6)	.06
eGFR, mean (SD), ml/min/1.73m2	66.4 (27.8)	66.3 (27.6)	.07
Proteinuria, median (IQR), mg/24h	1716 (695-3871)	1560 (500-3544)	.35
Hemoglobin, mean (SD), g/dL	12.3 (2.3)	12.3 (2.3)	.10
LVMI, median (IQR), g/m²	85.3 (73.5-103.6)	80.5 (70.6-95.2)	.06
BP parameter			
Office SBP, mean (SD), mmHg	129.5 (8.5)	126.5 (8.1)	.46
Office DBP, mean (SD), mmHg	80.1 (6.7)	76.4 (6.2)	.43
24-h SBP, mean (SD), mmHg	134.8 (9.1)	121.2 (7.7)	.26
24-h DBP, mean (SD), mmHg	86.2 (6.3)	76.8 (5.1)	.53
Daytime SBP, mean (SD), mmHg	135.6 (8.2)	121.3 (7.5)	.49
Daytime DBP, mean (SD), mmHg	87.0 (6.3)	77.0 (5.4)	.89
Nighttime SBP, mean (SD), mmHg	130.2 (17.2)	120.6 (12.0)	.01
Nighttime DBP, mean (SD), mmHg	81.8 (9.2)	76.1 (6.7)	<.001
SBP dipping rate, mean (SD), %	4.1 (8.9)	0.6 (7.8)	.27
DBP dipping rate, mean (SD), %	5.9 (8.7)	1.0 (8.1)	.54

© 2022 Fu X et al. *JAMA Network Open.*

Values are means (standard deviations), median (quartile ranges) or number (percentage). SI conversion factors: To convert fasting glucose to mmol/L, multiply by 0.0555; albumin to g/L, multiply by 10; total cholesterol to mmol/L, multiply by 0.0259; serum creatinine to μmol/L, multiply by 88.4; hemoglobin to g/L, multiply by 10.

Abbreviations: CKD, chronic kidney disease; ACEI, angiotensin-converting enzyme inhibitor; ARB, angiotensin receptor blocker; CCB, calcium channel blocker; BMI, body mass index; eGFR, estimated glomerular filtration rate; Cr, creatinine; eGFR, estimated glomerular filtration rate; LVMI, left ventricular hypertrophy index; SBP, systolic blood pressure; DBP, diastolic blood pressure. MUCH, masked uncontrolled hypertension

© 2022 Fu X et al. *JAMA Network Open.*

eTable 6. Association Between Left Ventricular Hypertrophy and Stage 1-2 Masked Uncontrolled Hypertension

Hypertension Types	No. LVH/No. Patients (%)	Unadjusted		Model 1		Model 2	
		OR (95% CI)	*P* value	OR (95% CI)	*P* value	OR (95% CI)	*P* value
Controlled Hypertension	6/115 (5.2)	1 [Reference]	NA	1 [Reference]	NA	1 [Reference]	NA
MUCH Stage 1	25/178 (14.0)	3.07 (1.22-7.74)	.02	2.89 (1.14-7.35)	.02	2.29 (0.87-6.05)	.09
MUCH Stage 2	14/66 (21.2)	5.19 (1.88-14.31)	.001	5.05 (1.82-14.02)	.002	4.03 (1.38-11.79)	.01

Logistics regression analysis in patients with controlled hypertension, stage 1-2 MUCH.

Model 1: Adjustment variables include age, sex, and body mass index;

Model 2: Adjustment variables include age, sex, body mass index, diabetes mellitus, use of angiotensin-converting enzyme inhibitors/angiotensin receptor blockers, eGFR, hemoglobin, and proteinuria.

Abbreviations: MUCH, masked uncontrolled hypertension; CI, confidence interval; OR, Odds ratio.

© 2022 Fu X et al. *JAMA Network Open.*

eTable 7. Left Ventricular Mass Index among Different Etiology of Chronic Kidney Disease Patients

	Diabetic Nephropathy	Hypertensive Nephropathy	Primary Glomerulonephritis	Others	*P* value
Unadjusted, g/m^2	98.0 (99.4-117.3)	112.8 (105.8-120.0)	86.2 (83.5-88.9)	92.3 (88.5-96.2)	<.001
Adjusted, g/m^2	98.0 (89.0-10)	110.0 (103.0-117.0)	88.5 (85.9-91.1)	90.3 (86.6-94.1)	<.001

Values are mean (95% confidence interval). Adjustment variables include age, sex, body mass index, diabetes mellitus, use of angiotensin-converting enzyme inhibitors/angiotensin receptor blockers, eGFR, hemoglobin, and proteinuria.

© 2022 Fu X et al. *JAMA Network Open.*

eTable 8. Left Ventricular Hypertrophy Among Study Patients Additionally Adjusted by Etiology of Chronic Kidney Disease

	OR (95% CI)	*P* value
Controlled Hypertension	1 [Reference]	NA
MUCH	3.16 (1.26-7.95)	.01
Sustained Hypertension	4.85 (1.93-12.18)	.001
Controlled Hypertension	1 [Reference]	NA
Isolated nighttime MUCH	2.23 (0.79-6.26)	.13
Day-night MUCH	3.35 (1.13-9.95)	.03

Logistics regression analysis in patients with hypertension subtypes and MUCH subtypes. Adjustment variables include age, sex, body mass index, diabetes mellitus, etiology of CKD, use of angiotensin-converting enzyme inhibitors/angiotensin receptor blockers, eGFR, hemoglobin, and proteinuria.

Abbreviations: MUCH, masked uncontrolled hypertension; CI, confidence interval; OR, Odds ratio.

© 2022 Fu X et al. *JAMA Network Open.*

eTable 9. Composite Kidney Outcomes Among Study Patients Additionally Adjusted by Etiology of Chronic Kidney Disease

	HR (95% CI)	*P* value
Controlled Hypertension	1 [Reference]	NA
MUCH	3.16 (1.32-7.57)	.01
Sustained Hypertension	5.43 (2.29-12.87)	< .001
Controlled Hypertension	1 [Reference]	NA
Isolated nighttime MUCH	3.85 (1.52-9.80)	.005
Day-night MUCH	2.16 (0.77-6.08)	.14

Cox regression analysis in patients with hypertension subtypes and MUCH subtypes.

Adjustment variables include age, sex, body mass index, diabetes mellitus, etiology of Chronic Kidney Disease, use of angiotensin-converting enzyme inhibitors/angiotensin receptor blockers, eGFR, hemoglobin, and proteinuria.

Abbreviations: MUCH, masked uncontrolled hypertension; CI, confidence interval; HR, hazard ratio.

© 2022 Fu X et al. *JAMA Network Open.*

eTable 10. Association Between Composite Kidney Outcomes and Stage 1-2 Masked Uncontrolled Hypertension

Hypertension Types	No. Events/No. Patients (%)	Unadjusted		Model 1		Model 2	
		OR (95% CI)	*P* value	OR (95% CI)	*P* value	OR (95% CI)	*P* value
Controlled Hypertension	6/125 (5.2)	1 [Reference]	NA	1 [Reference]	NA	1 [Reference]	NA
MUCH Stage 1	29/178 (16.3)	4.20 (1.74-10.14)	.001	4.36 (1.78-10.66)	.001	3.44 (1.37-8.64)	.009
MUCH Stage 2	11/66 (16.7)	3.75 (1.39-10.15)	.009	3.72 (1.36-10.16)	.01	3.07 (1.10-8.58)	.03

Cox regression analysis in patients with controlled hypertension, stage 1-2 MUCH.

Note: Model 1: Adjustment variables include age, sex, and body mass index;

Model 2: Adjustment variables include age, sex, body mass index, diabetes mellitus, use of angiotensin-converting enzyme inhibitors/angiotensin receptor blockers, eGFR, hemoglobin, and proteinuria.

Abbreviations: MUCH, masked uncontrolled hypertension; CI, confidence interval; HR, hazard ratio.

© 2022 Fu X et al. *JAMA Network Open.*

eTable 11. Association Between Left Ventricular Hypertrophy and Hypertension Subtypes Based on 2017 ACC/AHA Hypertension Guidelines

Hypertension Types	No. Events/No. Patients (%)	Unadjusted		Model 1		Model 2	
		OR (95% CI)	*P* value	OR (95% CI)	*P* value	OR (95% CI)	*P* value
Controlled Hypertension	3/74 (4.0)	1 [Reference]	NA	1 [Reference]	NA	1 [Reference]	NA
MUCH	11/107 (10.3)	2.62 (0.70-9.76)	.15	2.68 (0.72-10.06)	.14	2.10 (0.55-8.07)	.28
Sustain Hypertension	101/440 (23.0)	6.78 (2.08-22.02)	.001	6.78 (1.99-21.61)	.002	4.68 (1.39-15.76)	.01

Logistics regression analysis in patients with controlled hypertension, MUCH and sustained hypertension.

Model 1: Adjustment variables include age, sex, and body mass index;

Model 2: Adjustment variables include age, sex, body mass index, diabetes mellitus, use of angiotensin-converting enzyme inhibitors/angiotensin receptor blockers, eGFR, hemoglobin, and proteinuria.

Abbreviations: ACC/AHA, American College of Cardiology/American Heart Association; MUCH, masked uncontrolled hypertension; CI, confidence interval; OR, Odds ratio.

© 2022 Fu X et al. *JAMA Network Open.*

eTable 12. Association Between Composite Kidney Outcomes and Hypertension Subtypes Based on 2017 ACC/AHA Hypertension Guidelines

Hypertension Types	No. Events/No. Patients (%)	Unadjusted		Model 1		Model 2	
		OR (95% CI)	*P* value	OR (95% CI)	*P* value	OR (95% CI)	*P* value
Controlled Hypertension	2/74 (2.7)	1 [Reference]	NA	1 [Reference]	NA	1 [Reference]	NA
MUCH	16/107 (15.0)	6.25 (1.44-27.22)	.02	6.45 (1.47-28.18)	.01	4.78 (1.09-21.03)	.04
Sustain Hypertension	111/440 (25.2)	12.11 (2.99-49.10)	<.001	13.70 (3.34-56.16)	<.001	6.73 (1.63-27.86)	.01

Cox regression analysis in patients with controlled hypertension, MUCH and sustained hypertension.

Model 1: Adjustment variables include age, sex, and body mass index;

Model 2: Adjustment variables include age, sex, body mass index, diabetes mellitus, use of angiotensin-converting enzyme inhibitors/angiotensin receptor blockers, eGFR, hemoglobin, and proteinuria.

Abbreviations: ACC/AHA, American College of Cardiology/American Heart Association; MUCH, masked uncontrolled hypertension; CI, confidence interval; HR, hazard ratio.

Figure legends:

eFigure 1. Association Between ESKD and Hypertension Subtypes.

A: Cox regression analysis in patients with controlled hypertension, MUCH and sustained hypertension. B: Cox regression analysis in patients with controlled hypertension, isolated nighttime MUCH and day-night MUCH.

Model 1: Adjustment variables include age, sex, and body mass index.

Model 2: Adjustment variables include age, sex, body mass index, diabetes mellitus, use of angiotensin-converting enzyme inhibitors/angiotensin receptor blockers, eGFR, hemoglobin, and proteinuria.

Abbreviations: MUCH, masked uncontrolled hypertension; CI, confidence interval; HR, hazard ratio.

© 2022 Fu X et al. *JAMA Network Open.*

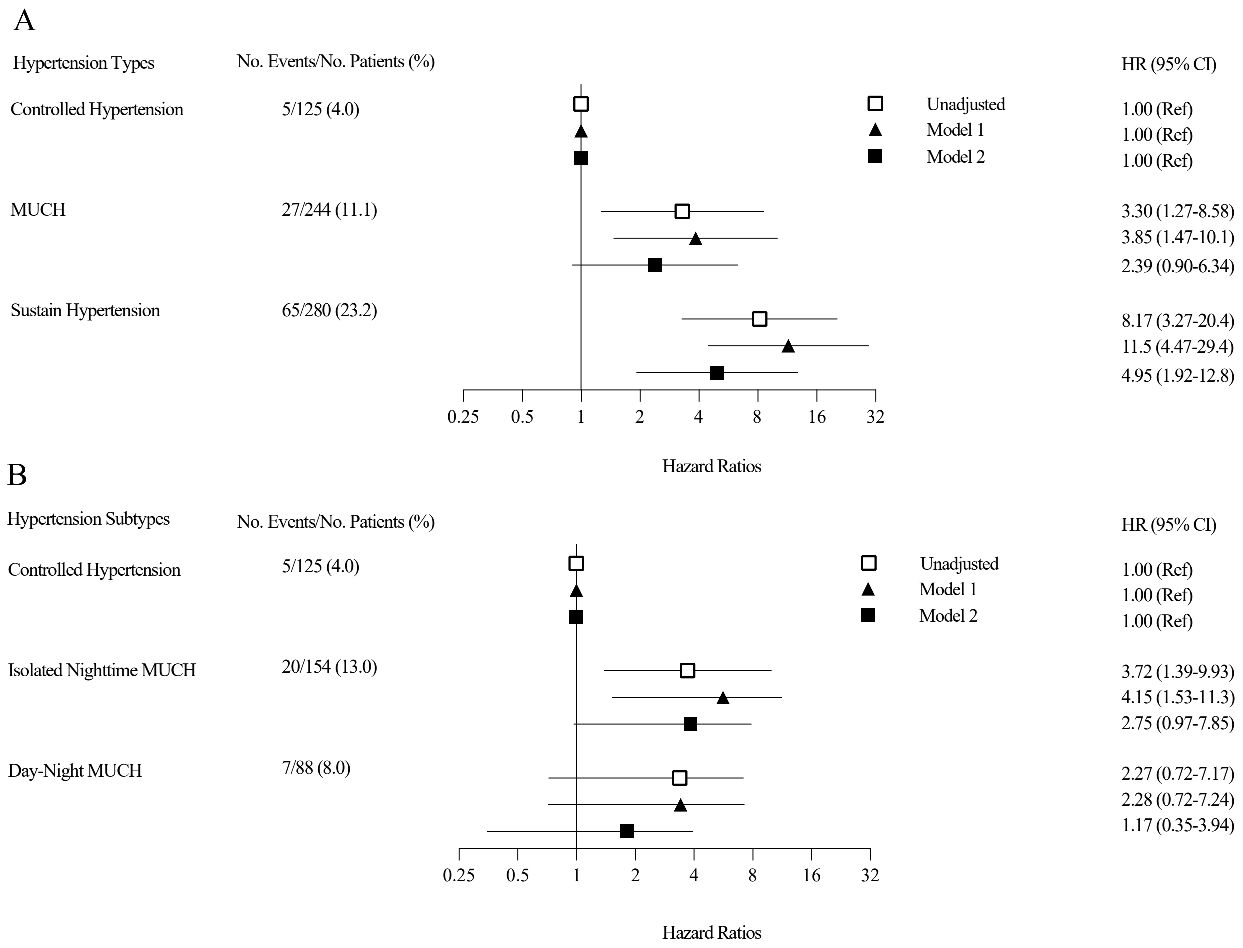

© 2022 Fu X et al. *JAMA Network Open.*
